# Supplementary material for: Human Serum, Following Absorption of Fish Cartilage Hydrolysate, Promotes Dermal Fibroblast Healing through Anti-Inflammatory and Immunomodulatory Proteins
Source: Biomedicines. 2024 Sep 19;12(9):2132. doi: 10.3390/biomedicines12092132 (PMC11430497; doi:10.3390/biomedicines12092132)
Supplement: Supplementary file 1 [file biomedicines-12-02132-s001.zip › biomedicines-3126335-supplementary/File S1_M&M.pdf]

2. Materials and methods

2.2. Experimental design

2.2.1. Ethics and Clinical Trial

The clinical part of the protocol was approved by the French Ethical Committee (2021-ND77 RIPH2 HPS/N° SI RIPH: 21.01436.000014/N° EudraCT/ID RCB: 2021-A01773-38/Comité de Protection des Personnes CPP Paris, Ile-de-France 1; approved 08 October 2021). The investigation was conducted with respect regarding the Declaration of Helsinki of 1975 (https://www.wma.net/what-we-do/medical-ethics/declaration-of-helsinki) revised in 2013. All volunteers were informed about the objectives and the potential risks of the investigation. Their written informed consent was obtained before their recruitment.

2.2.2. Human Study protocol

The human *ex vivo* methodology used in this study has been registered as a written invention disclosure by the French National Institute for Agronomic, Food and Environment Research (INRAE) (DIRV#18-0058). Clinic'n'Cell® has been registered as a mark.

A pool of 10 healthy men (age: 25.4 years old, +/−3.7; BMI: 23.6 kg/m2, +/−1.9; >60 kg; without drug treatment; and no distinction on ethnicity) was recruited for this study. Before participation, all volunteers were checked for normal ranged blood formulation, renal (urea and creatinine) and liver functions (aspartate

aminotransferase (AST), alanine aminotransferase (ALT), gamma-glutamyltransferase (GGT) activities) to ensure volunteer safety and accurate data collection. Blood samples were performed, teched, aliquoted and stored at the Centre d'Investigation Clinique de Clermont-Ferrand - Inserm U1405. The clinical part comprised two phases. The first one was dedicated to validate and determine the human FCH metabolites absorption profile. Ten fasted volunteers received 12 g of FCH and were subjected to blood sampling. Nine mL of venous blood were collected before the ingestion and every 20 min for 240 min after the ingestion. Serum was separated from blood cells and immediately stored at −80 °C until further analyzed. Hydrolyzed collagen peptides and CS absorption profiles were evaluated by ELISA targeting hydroxyproline and CS, respectively. The analyses showed that following the ingestion of FCH, circulating concentrations of hydroxyproline and CS reach a maximum at 140 min post-ingestion. The second phase was set to collect both naive and FCH-enriched serum fractions. As for the first step, 10 healthy volunteers fasted for 12 h and were then given 12 g of FCH. A total of 48 mL of venous blood was collected before the ingestion for naive (baseline) serum collection. Then, at the maximum absorption peak (140 min post-ingestion), 48 mL of blood as well was collected for metabolites-enriched serum collection. Serum was stored at −80 °C until analysis.

2.5. DiaPASEF proteomic analysis

2.5.2. Nanoliquid chromatography coupled with tandem mass spectrometry (NanoLC-MS/MS) acquisition of fractions for spectral library – DDA-PASEF

All fractions (n = 12) of the different pools were analysed in Data-Dependent Analysis (DDA) and PASEF mode to generate the spectral library. Each fraction of enzymatically digested proteins (about 200 to 300 ng) was separated onto a 75 µm × 250 mm IonOpticks Aurora 3 C18 column (Ion Opticks Pty Ltd., Bundoora, Australia). A gradient of reversed phase buffer (Buffer A: 0.1% formic acid, 2% acetonitrile, 97.9% H2O; Buffer B: 0.1% formic acid, 99,9% acetonitrile) was run on a nanoElute UHPLC System (Bruker Daltonik GmbH, Bremen, Germany) at a flow rate of 250 nL/min at 50 ◦C controlled by HyStar software (v6.0.30.0, Bruker Daltonik). The liquid chromatography (LC) run lasted for 80 min. A starting concentration of 2% buffer B increasing to 13% over the first 42 min was first performed and buffer B concentrations were increased up to 20% at 65 min; 30% at 70 min; 85% at 75 min and finally 85% for 5 min to wash the column. The temperature of the ion transfer capillary was set at 180 ° C. Ions were accumulated for 100 ms, and mobility separation was achieved by ramping the entrance potential from −160 V to −20 V within 114 ms. The acquisition of the MS and MS/MS mass spectra was done with average resolutions 50.000 FWHM full width at half maximum (mass range 100–1700 m/z), respectively. To enable the PASEF method, precursor m/z and mobility information was first derived from full scan TIMS-MS experiments (with a mass range of m/z 100–1700). The quadrupole

isolation width was set to 2 and 3 Th and, for fragmentation, the collision energies varied between 31 and 52 eV depending on the precursor mass and charge. TIMS, MS operation and PASEF were controlled and synchronized using the control instrument software OtofControl 6.2.5 (Bruker Daltonik). LC-MS/MS data were acquired using the PASEF method as previously described, with a total cycle time of 1.31 s, including 1 TIMS MS scan and 10 PASEF MS/MS scans. The 10 PASEF scans (100 ms each) contained, on average, 12 MS/MS scans per PASEF scan. Ion mobility-resolved mass spectra, nested ion mobility vs. m/z distributions, as well as summed fragment ion intensities were extracted from the raw data file with DataAnalysis 6.0 (Bruker Daltonik).

2.5.3. NanoLC-MS/MS acquisition of samples in diaPASEF mode

Twenty samples were then analysed individually in diaPASEF mode. Each tryptic peptide sample, of approximately 400-500 ng each, was analysed under the conditions described above. These included the same analytical conditions (identical instrumentation, type of separation column and gradient length) and analysis on the same instrument (timsTOF Pro, Bruker Daltonik). For the development of the diaPASEF method, a method with an adapted instrument firmware was used, to perform data-independent isolation of data from several 25 m/z wide precursor windows, also called segments, in a single TIMS separation (107.5 ms). A method with two boxes per segment was used in each 107.5 ms diaPASEF scan, *i.e.*, a total of thirty-

two segments and sixty-four boxes, of which sixteen of these scans perfectly cover the diagonal area of doubly and triply charged peptides in the m/z and ion mobility output range. MS and MS/MS data were collected over the m/z range 100 - 1700 and over the mobility range from 1/K0 = 0.6 to 1/K0 = 1.6 Vs cm-2. During each data collection, each TIMS cycle was 1.25 seconds long and comprised 1 MS and 22 cycles of diaPASEF MS/MS segments, comprising 2, 3 or 4 boxes, to cover a total of 64 boxes defined in the acquisition method. The collision energy was increased linearly with mobility from 68 eV at 1/K0 = 1.6 Vs cm^{-2} to 25 eV at 1/K0=0.6 Vs cm^{-2}.
