# Supplementary figures and images for: Human Serum, Following Absorption of Fish Cartilage Hydrolysate, Promotes Dermal Fibroblast Healing through Anti-Inflammatory and Immunomodulatory Proteins
Source: Biomedicines. 2024 Sep 19;12(9):2132. doi: 10.3390/biomedicines12092132 (PMC11430497; doi:10.3390/biomedicines12092132)

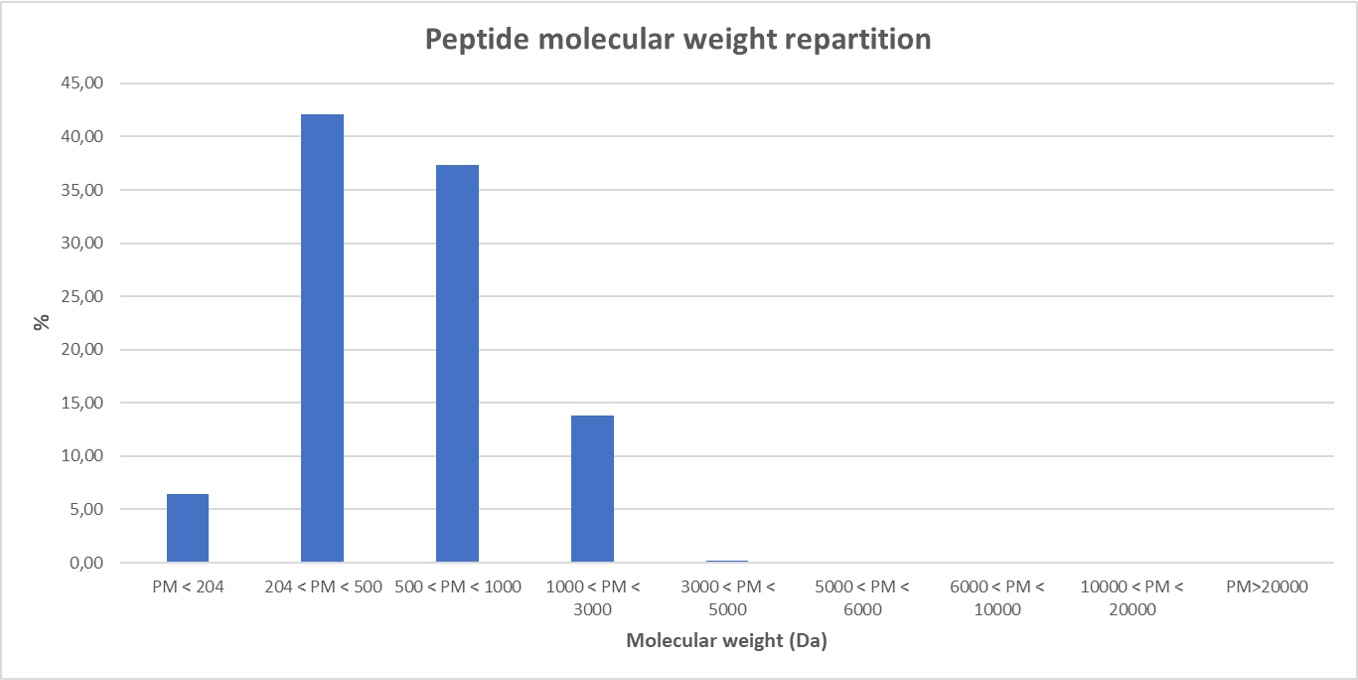

Supplement: Supplementary file 1 [file biomedicines-12-02132-s001.zip › biomedicines-3126335-supplementary/Figure S1.jpg]
